# Squid – a simple bioinformatics grid

**DOI:** 10.1186/1471-2105-6-197

**Published:** 2005-08-03

**Authors:** Paulo C Carvalho, Rafael V Glória, Antonio B de Miranda, Wim M Degrave

**Affiliations:** 1 Laboratory for Functional Genomics and Bioinformatics, Oswaldo Cruz Institute, Fiocruz, Rio de Janeiro, Brazil

## Abstract

**Background:**

BLAST is a widely used genetic research tool for analysis of similarity between nucleotide and protein sequences. This paper presents a software application entitled "Squid" that makes use of grid technology. The current version, as an example, is configured for BLAST applications, but adaptation for other computing intensive repetitive tasks can be easily accomplished in the open source version. This enables the allocation of remote resources to perform distributed computing, making large BLAST queries viable without the need of high-end computers.

**Results:**

Most distributed computing / grid solutions have complex installation procedures requiring a computer specialist, or have limitations regarding operating systems. Squid is a multi-platform, open-source program designed to "keep things simple" while offering high-end computing power for large scale applications. Squid also has an efficient fault tolerance and crash recovery system against data loss, being able to re-route jobs upon node failure and recover even if the master machine fails. Our results show that a Squid application, working with N nodes and proper network resources, can process BLAST queries almost N times faster than if working with only one computer.

**Conclusion:**

Squid offers high-end computing, even for the non-specialist, and is freely available at the project web site. Its open-source and binary Windows distributions contain detailed instructions and a "plug-n-play" instalation containing a pre-configured example.

## Background

Bioinformatics includes some highly repetitive and computing intensive applications, such as comparison of nucleotide or peptide sequences in search for similarities. The BLAST algorithm (Basic Local Alignment and Search Tool) is well known for its performance [1]. Even though BLAST is "fast", it is an increasingly time-consuming operation when many sequences are to be queried against large databases.

Web pages offering BLAST capabilities are limited in the number of query sequences and available databases to search, while local facilities can easily get overloaded due to limited computing resources when dealing with data intensive operations in smaller research centers. Grid computing permits usage of idle resources and is an inexpensive alternative to large multiprocessor machines or dedicated clusters.

"A computational grid is a hardware and software infrastructure that provides dependable, consistent, pervasive and inexpensive access to high-end computational capabilities" [2]. There are high-quality grid initiatives such as Globus [3], N1 Grid Engine 6, MyGrid [4] and EUROGRID [5] among others. Such software acts as middleware interlinking resources of multiple computers within or between institutions using open and general purpose protocols for secure high performance distributed computing. Although some of these solutions are very complete, their installation is quite complex, and may require specialist management, or have limitations regarding the operating system. Currently, a few non-commercial packages specialized in performing distributed BLAST are available. Among them stand out S-BLAST [6], BeoBlast [7], CondorBLAST [8], Soap-HT-BLAST [9], W.ND BLAST [10], and mpiBLAST [11]. However, only SOAP- HT-BLAST and W.ND BLAST present GUIs, and the latter is only Windows based. Thus a few drawbacks for these solutions are: GUI absence, the need for extensive networking skill for implementation, low fault tolerance [10] and not being multi-platform, resulting in limited utility for the end-user.

In this work a simple, low-cost, platform independent, easy to install and use computational grid interface was constructed and applied to BLAST implementation. This environment called Squid is based on TCP/IP [12], automatically manages available computing resources and makes large BLAST queries possible, even for small laboratories with limited computing resources.

## Implementation

The Squid environment is programmed in Perl (version 5.8.6) and is composed of two programs, Squid and Tentacle, each having their configuration file in text format, which can be edited by the user to reflect the local configuration. The graphic user interface (GUI – images can be seen at project web site) can be used to view the currently available computing resources, select query sequence files and choose databases to confront. Squid can also be fully controlled through the command prompt allowing experienced users to encapsulate it within other programs and pipelines. Each grid node must have a copy of the BLAST and Tentacle programs installed and, ideally, a copy of the database. Although remote DB copies can be accessed, this can heavily overburden the connecting network and degrade performance. Squid can be installed under Linux, Unix, Mac OS and Windows operating systems. As soon as the user submits a job, Squid will test which of the nodes are up and properly configured to receive jobs. Squid also timely checks the availability of new up-nodes.

The user-submitted file containing the query sequences is split into smaller files called "work fragments" with user preconfigured size (ex. a file with 10000 query sequences can be split in 200 files with 50 query sequences each), and kept in a work directory. By knowing the up time and availability of nodes, fragment size can be adapted to best suit various working environments. Each fragment is sent to an available node for BLAST execution. Only when the BLAST results file is successfully returned, the respective fragment file is removed from the work directory. Squid will continue to send jobs to nodes until there are no more fragments located in the work directory. This approach is a simple yet highly efficient job control and crash recovery system.

Tentacle is responsible for a single nodes internal administration. The communication semantics consists in receiving a command, (i.e. blast, reset_node, erase_work_files, authenticate, etc.), followed by required complementary data. The command is validated, processed and an answer is always sent back to the central administration node running Squid.

Squids' remote node administration core works by managing three lists: the up_node (nodes that are ready to receive work), down_node (nodes that are not responding), and busy_node (nodes that are currently processing). After creation of the "work fragments" in the work directory, node classification as belonging to the up_node or down_node list is performed. This is quickly accomplished by sending preset messages to every remote node and validating if the proper response is returned, much like the *ping *command in most operational systems.

Successfully validated nodes are added to the up_node list. Subsequently, "work fragment" files are selected from the work directory and sent to nodes in the up_node list, together with the blast command. The receiving nodes are moved from the up_node list to the busy_node list. Squid continues to send jobs and manage them in different threads until no more nodes are available in the up_node list. When a BLAST result is fully received from a remote node, it is written to disk and its corresponding work fragment is then deleted from the work directory. Finally the node is changed from the busy_node to the up_node list. It is important to note that a small overhead occurs for every communication established, therefore larger work fragments can result in less overhead.

To check if processing nodes are still up, validation commands are timely sent. If validation fails, indicating that the node has stopped serving the grid, it is immediately removed from the up_node or busy_node list and placed in the down_node list. A thread timely sends validation requests to nodes in the down_node list. If the node responds again, a node reset command is sent, clearing local work files, and it is again moved to the up_node list. The "lost fragment" will be re-routed to a new node, since Squid only erases the "work fragment" from the work directory after receiving and saving its complete BLAST result.

A fault recovery routine is also implemented to handle occasions where the main node goes down. Before reactivating Squid, the crash recovery button in the GUI can be clicked. This makes Squid jump the routine where the initial FASTA file is read and fragmented into "work fragments" that are placed in the work directory; thus only the work fragments that haven't been processed will remain. Squid will once again go through node validation and pick up right from where it left.

Data can be lost in intermediate networks or in unstable connections, but the TCP/IP communication protocol is capable of detecting such errors and automatically trigger retransmission until data is correctly and completely received. Squid's node communication is fully based on TCP/IP addressing and data transfer verification through a user configured port. Even though Squid authenticates a remote node before receiving input, enhanced security can be obtained by setting up a virtual private network (VPN) when working across unsafe networks. A VPN performs data tunnelling (making sure that it cannot be intercepted) and encryption; linking it with Squid should guarantee a secure and reliable data transmission, especially when sensitive data is involved. Since there is always a loss in performance when using encrypted data, such degree of safety should be evaluated.

## Results and conclusion

Squid is designed to "keep things simple" offering grid power for large scale applications (BLAST in the current configuration) for smaller labs, so the user gets to worry about analyzing results while Squid worries about distributed computing and getting the job done. Squid stands out among other software because it can simultaneously work with Windows / Linux nodes and efficiently manage job control but above all, it is meant to be user friendly. One can subsequently use BioParser, also available at our lab page to further process large BLAST outputs. Various tests were carried out to analyze Squids' performance and robustness where the following should be noted:

1. A grid containing N nodes is able to execute multiple BLAST queries almost N times faster than if working with only one node.

2. Overhead occurs due to computer communication, network latency and initiating new computer processes. Thus the size of fragment files should not be too small.

3. For maximum performance, each node should have a copy of the database, but remote copies can also be used.

4. Squid successfully handles problems such as unexpected remote node shutdown or even main node accidental shut down. Squid picks up right from were it left.

Every time a node is available for a job, Squid sends it a work fragment. If the node stops serving the grid while processing, its uncompleted work file will be eliminated and Squid will eventually re-route the "skipped fragment" to another available node. Being so, by knowing the up time and availability of nodes, fragment size can be adapted to best suit for various working environments.

Further help and instructions are included within the distribution. Open source and binary Windows versions also come with a pre-configured example for evaluation purposes.

## Availability and requirements

• **Project name: **Squid – A simple bioinformatics grid

• **Project home page: **Squid is available for download at the projects website [13]. Once in the site select softwares and Squid to view the project page and download links.

• **Operating system(s): **Platform independent

• **Programming language: **Perl 5.8.6

• **License: **Creative Commons – Commons Deed [14].

• **Any restrictions to use by non-academics: **license needed

## Authors' contributions

PCC performed software engineering, coding, elaboration of manuscript. RVG, ABM and WD performed software testing, benchmarks, helped in GUI coding, debugging and manual / manuscript revisions.
